# Outlier detection of skill decision differences of football penalty takers from a data-driven perspective: multialgorit hm modeling and empirical research of eye-tracking data

**DOI:** 10.3389/fspor.2026.1733178

**Published:** 2026-08-04

**Authors:** Zheng Li, Pu Hu, Hua Liu, Ruo Chen Dai

**Affiliations:** 1 Wuhan Sports University, School of Intelligent Sports Engineering, Wuhan, Hubei, China; 2 Wuhan Sports University, Football Performance Analysis Laboratory, Wuhan, Hubei, China; 3 Wuhan Sports University, School of Sports Medicine, Wuhan, Hubei, China; 4 Wuhan Sports, University Football Academy, Wuhan, Hubei, China

**Keywords:** DBSCAN clustering, expert-novice paradigm, eye movement tracking, machine learning, outlier detection, penalty taker

## Abstract

Defensive performance in football penalty kicks relies heavily on a goalkeeper's visual anticipation and rapid decision-making capabilities. While identifying the visual search advantages of expert athletes is crucial for optimizing training, conventional eye-tracking metrics often fail to capture the complex spatial distribution patterns of gaze points. Integrating machine learning anomaly detection algorithms offers a novel methodological perspective for the precise quantification and analysis of visual strategy variations across different expertise levels.

**Objective:**

This study aimed to investigate the differences in visual search characteristics between expert and amateur football players during penalty defense tasks, leveraging eye-tracking technology and multiple machine learning algorithms.

**Methods:**

Gaze data were collected from 10 professional/semi-professional athletes (Expert Group) and 10 collegiate athletes (Amateur Group), resulting in 120 valid records. The Shapiro–Wilk test indicated that the gaze point coordinates (*X* and *Y* axes) were not normally distributed. Consequently, four machine learning anomaly detection algorithms—Density-Based Spatial Clustering of Applications with Noise (DBSCAN), Local Outlier Factor (LOF), Isolation Forest, and Elliptic Envelope—were employed to perform a systematic outlier analysis on the gaze coordinates.

**Results:**

(1) DBSCAN results revealed a highly significant difference in outlier rates between the two groups (p=0.0053<0.01). The outlier rate for the Expert Group (53.57%) was significantly lower than that of the Amateur Group (61.27%), with a medium effect size $\left(\mathrm{Cohe}n^{\prime }s\;d=0.46\right)$. (2) Under a 5% contamination rate setting, no significant differences were detected by the LOF, Isolation Forest, or Elliptic Envelope algorithms (p>0.05). (3) Composite analysis showed that the proportion of points identified as outliers by at least one algorithm differed significantly between groups (Expert: 54.47% vs. Amateur: 62.04%, p=0.0052), representing a practically meaningful difference $\left(\mathrm{Cohe}n^{\prime }s\;d=0.47\right)$.

**Conclusion:**

Expert athletes exhibit more concentrated and stable visual search strategies (characterized by lower gaze outlier rates) during penalty defense compared to amateurs. The DBSCAN algorithm and composite indicators effectively quantify expertise-related visual search features, providing empirical evidence for understanding cognitive patterns and enhancing scientific training for football goalkeepers.

## 1 Introduction

As the world's number one sport, the outcome of football matches is often determined by crucial moments. Penalty kicks, as one of the most dramatic elements in football matches, place extremely high demands on the penalty taker's reaction ability, anticipation ability and psychological quality (1). The penalty taker must complete the judgment of the goalkeeper's intention to save and make a penalty kick within a short period of time. This process is highly dependent on the rapid extraction and processing of visual information (2).

Eye-tracking technology provides an objective quantitative means for exploring the visual cognitive processing of athletes (3). By recording the spatio-temporal distribution characteristics of the staring points of the eyes, researchers can reveal the essential differences between experts and novices in visual search strategies, attention allocation, and information extraction efficiency (4). Traditional studies mostly employ descriptive statistical indicators such as the number of fixation points, fixation duration, and slitting amplitude, but these methods are difficult to comprehensively depict the spatial distribution patterns and abnormal characteristics of eye movement data (5). The “Quiet Eye” (QE) theory proposed by Vickers (6) is a milestone in the study of motor eye movement. This theory indicates that expert athletes have a longer duration of final fixation before performing key movements. Existing research mainly focuses on the visual search strategies of penalty takers, exploring the distribution characteristics of their fixation points in save decisions. Some studies have found that expert groups tend to focus more on the core areas of the opponent's body, such as the trunk and hips, thereby enhancing the accuracy of their predictions. In contrast, the gaze points of the amateur group were more scattered, mostly concentrated on peripheral cues such as the limbs, and were easily affected by interfering information (7). This difference further supports the H3 hypothesis, indicating that experts have developed an efficient information extraction model through long-term training. In addition, experts demonstrate stronger fixation stability and predictive scanning capabilities in the dynamic decision-making process, enabling them to identify key movement cues earlier and reduce fixation disturbances in irrelevant areas. However, amateurs, due to their lack of experience, often experience frequent backtracking and disordered jumping, which leads to an increase in visual noise and supports the inference about the difference in outlier rates in Hypothesis H1. By integrating the application of anomaly detection algorithms, these eye movement features can be quantified as outlier density and distribution patterns, further revealing the perception mechanism behind the expert's advantages. In some studies on goalkeepers' penalty saves, Savelsbergh et al. (2, 8) found that expert goalkeepers paid more attention to the non-ball side foot and hip area of the penalty taker, while novice goalkeepers overly focused on the ball itself. This verifies the necessity of studying the characteristics of the visual search strategy for penalty takers. The research of Williams et al. (1) further indicates that experts gain an advantage through “anticipation” (anticipation) rather than merely reaction speed, and this anticipation ability is highly correlated with specific visual search patterns.

The Outlier Detection algorithm in machine learning provides a new perspective for eye movement data analysis (9). Anomaly/Outlier Detection is an important branch in machine learning, aiming to identify samples in data that deviate from the normal pattern (9). The DBSCAN (density-based Spatial Clustering of Applications with Noise) algorithm proposed by Ester et al. (10) identifies core points, boundary points and noise points by defining neighborhood Density. Its core parameters include the neighborhood radius *ε* and the minimum sample size MinPts. This algorithm does not require presetting the number of clusters, has strong robustness to noise, and is particularly suitable for datasets with spatial distribution characteristics (11). The Local Outlier Factor (LOF) algorithm proposed by Breunig et al. (12) quantifies the degree of anomaly by comparing the local densities of sample points and their neighborhoods, and is capable of discovering local density anomaly points. Liu et al.'s (13) Isolation Forest is based on the assumption that “outliers are more likely to be isolated”, and achieves efficient global anomaly detection through random tree partitioning. Rousseeuw and Driessen (7) assumed that normal data followed a multivariate Gaussian distribution and identified points deviating from the distribution center through robust estimation of the covariance matrix. These algorithms have achieved successful applications in fields such as financial fraud detection (13), network intrusion detection (14), and medical diagnosis (15). However, its application in eye movement data analysis is still in its infancy.

Chase and Simon (16) established the expert-novice comparison paradigm in their pioneering research and found that experts efficiently encode and extract domain knowledge through the “chunking” strategy. In the field of sports, Abernethy (17) systematically reviewed the advantages of expert athletes in spatio-temporal occlusion tasks, pointing out that experts are better at extracting predictive information from early kinematic cues. Mann et al.'s (4) meta-analysis integrated 42 eye movement studies and found that expert athletes exhibited the following visual search characteristics: (1) less but longer fixation; (2) Pay more attention to body parts that are rich in information; (3) Start searching for key clues earlier. The “perception-motor workspace” theory proposed by North et al. (18) emphasizes that the perceptual skills and motor skills formed by experts through long-term training are closely coupled, and eye movement patterns are the external manifestations of this coupling relationship.

In conclusion, existing research has established the advantages of expert penalty takers in visual search, but the quantitative analysis of their spatial distribution characteristics is insufficient. Density-based clustering algorithms such as DBSCAN can identify outliers and noise in data, reflecting the concentration of visual search. Local density algorithms such as LOF can capture local abnormal patterns. Isolation Forest and Elliptic Envelope evaluate the degree of anomaly of data points from a global perspective. Applying these algorithms to the eye movement data analysis of penalty takers is expected to uncover expert-novice differences that are difficult to reveal by traditional methods (19), aiming to deepen the understanding of expert-novice differences from the perspective of data distribution.

## 2 Materials and methods

### 2.1 Experimental design

#### 2.1.1 Subject selection

This study recruited two groups of subjects: The expert group included 10 professional/semi-professional football players (with an average age of 24.3 ± 3.8 years and a training period of 8.7 ± 2.5 years), all of whom were players of national first-class or above professional teams; The amateur group consists of 10 college football players (with an average age of 21.8 ± 2.1 years and a training period of 2.3 ± 1.2 years), who possess basic penalty skills and are students majoring in football in the field of physical education teaching. All subjects had normal vision or corrected vision, no history of neurological diseases, voluntarily participated in the experiment and signed the informed consent form. The research was approved by the ethics committee of the institution where it was conducted (Authorization number: 2025127).

#### 2.1.2 Experimental equipment and materials

Eye movement data collection was carried out using the Tobii Pro Glasses 2 wearable eye tracker (sampling frequency 100 Hz, precision 0.5°, viewing Angle range 82° × 52°). The experimental site is the penalty area of a standard football field (16.5 meters wide). The goalkeeper stands in the center of the goal line, and the penalty taker is 11 meters away from the goal (penalty spot). The experimental material was a standard size 5 football, and the height and body type of the goalkeepers were similar to control the body type variable.

#### 2.1.3 Experimental procedure

The experiment adopted a random block design. Each penalty taker completed three sets of penalty shootout tasks, with each set consisting of three penalties (the speed and method of the penalties were randomly changed to control the confusatory variables). The goalkeeper is a trained standardized actor who performs kicking actions according to the preset plan to ensure consistency of the movements. The subjects wore eye trackers and stood in front of the standard penalty spot. The experimental instruction was “Please do your best to take the penalty kick as you would in a real game.” The eye tracker is calibrated at a single point (with a calibration error of less than 0.5°) before each set of tasks, and the eye movement data throughout the entire process from the start of the penalty taker's run-up to the moment the ball leaves the ground is recorded.

### 2.2 Data acquisition and preprocessing

The original eye movement data were exported by Tobii Pro Lab 1.194 software in Excel format (.xlsx) and tsv format (.tsv), including 433 variables such as timestamps, gaze point coordinates (*X*/*Y*), pupil diameter, validity markers, etc. The core variables of concern in this study are “Gaze point X [MCS px]” and “Gaze point YY [MCS px]”, representing the pixel coordinates of the gaze point in the Monitor Coordinate System.

The data preprocessing process includes: (1) deleting invalid samples for blinking and tracking lost moments (validity marked as 0 or NaN); (2) eliminate records that are missing both *X* and *Y* coordinates simultaneously; (3) detect and handle outliers that exceed the screen range (*X* < 0 or *X* > 1,920, *Y* < 0 or *Y* > 1,080); (4) calculate the valid sample size for each group of data and exclude low-quality records with valid samples less than 200. After pretreatment, the expert group retained 60 valid records (with an average of 2,461 ± 745 valid sample points per group), while the amateur group retained 60 valid records (with an average of 2,184 ± 682 valid sample points per group).

### 2.3 Anomaly detection algorithm

This study employs four anomaly detection algorithms to analyze the coordinates of eye movement gaze points.

#### 2.3.1 DBSCAN algorithm

DBSCAN (Density-Based Spatial Clustering Algorithm with Noise) defines cluster structures through density accessibility. The algorithm categorizes sample points into three types: core points, boundary points, and noise points. A core point is defined as a sample point *p* that contains at least Minpts sample points within its *ε*-neighborhood *Nε*(*p*). Specifically:

$$N_{\varepsilon }(p)=\{q\epsilon D|\mathrm{dist}(\;p,q)\leq \varepsilon$$

The notation dist(*p*,*q*) denotes the Euclidean distance between points *p* and *q*, with *D* representing the sample set. Boundary points are those located within the *ε*-neighborhood of a core point but failing to meet the core point criteria themselves.

This study set *ε* = 20 pixels (approximately corresponding to a 1.2-degree field of view) and Minpts = 20. Samples labeled as noise points (outliers) represent abnormal gaze points deviating from the primary focus area during visual search, reflecting the dispersion degree of visual search.

#### 2.3.2 Local outlier factor (LOF)

The LOF algorithm quantifies the degree of anomaly by comparing the local density between sample points and their neighboring regions. First, it calculates the reachability distance of the sample points:

$$\mathrm{reach}-\mathrm{dis}t_{k}(\;p,o)=\max\{k-\mathrm{distance}(o),d(\;p,o)\}$$

Local Reachability Density is defined as:

$$\mathrm{lr}d_{k}(p)=\frac{\left|N_{k}(p)\right|}{\sum_{o\in N_{k}(p)\mathrm{reach}-\mathrm{dis}t_{k}(\;p,o)}}$$

Here, *k* denotes the number of sample points within the neighborhood, and the LOF value reflects the degree of density deviation of point *p* relative to its neighborhood. In this study, *k* = 20 (neighborhood sample size) and contamination rate (contamination) = 0.05 were set, implying that 5% of samples are expected to be outliers. A LOF value >1 indicates that the sample density is lower than that of its neighborhood, with higher values indicating greater abnormality.

#### 2.3.3 Isolation forest

Isolation Forest is based on the hypothesis that “anomalies are more likely to be isolated”. It constructs isolation trees through random forests. The algorithm randomly selects features and split points to recursively partition data. Anomalies, due to deviating from dense data regions, are isolated earlier and have shorter path lengths.

The anomaly score for sample x is calculated as:

$$s(x,n)=2^{-\frac{E[h(x)|}{c(n)}}$$

Here, *E*(*h*(*x*)) denotes the average path length of sample x across all trees, while *c*(*n*) represents the normalization factor for the average path length of binary search trees constructed from *n* samples. A value of *s*(*x*) closer to 1 indicates a more anomalous sample, whereas values closer to 0 signify a more normal sample. This study specifies 100 trees and a contamination rate of 0.05.

#### 2.3.4 Elliptic envelope

The Elliptic Envelope hypothesis assumes that normal data follows a multivariate Gaussian distribution, with the center and shape of the data fitted using robust covariance estimation (Minimum Covariance Determinant, MCD). The Mahalanobis distance from sample points to the center is used to identify outliers:

$$MD(x)=\sqrt{{\left(x-\mu \right)}^{T\sum^{-1}}(x-\mu )}$$

where *µ* denotes the robustly estimated mean vector and Σ represents the robust covariance matrix. Samples with MD exceeding the critical value of the chi-square distribution *X*^2^(2,0.95) are labeled as outliers. In this study, a contamination rate of 0.05 was set, and a random seed of 42 was used to ensure reproducibility of results.

### 2.4 Statistical analysis methods

Data analysis was performed using Python version 3.9 or earlier, with the following libraries employed: pandas 2.0.0 (for data processing), numpy 1.24.0 (for numerical computations), scikit-learn 1.4.0 (for machine learning algorithm implementation), scipy 1.11.0 (for statistical testing), and matplotlib 3.7.0 with seaborn 0.12.0 (for data visualization).

Due to the skewed distribution of the eye-tracking data's outlier rate, which violated the normality assumption (Shapiro–Wilk test *p* < 0.01), non-parametric testing methods were adopted. Between-group difference analysis utilized the Mann–Whitney *U* test (two-tailed), with a significance level of *α* set at 0.05.

The formula for calculating the Mann–Whitney *U* statistic is:

$$U=n\ln\;2+\frac{n_{1}(n_{1}+1)}{2}-R_{1}$$

Among them, $n_{1}$ and $n_{2}$ represent the sample sizes of two groups, and $R_{1}$ is the rank sum of the first group of samples. The effect size was used to assess the practical significance of the differences between groups using cohen' d:

$$d=\frac{M_{1}-M_{2}}{SD_{\mathrm{pooleed}}}$$

According to Cohen's criteria (1988), a value of *d* = 0.2 indicates a small effect, *d* = 0.5 indicates a moderate effect, and *d* = 0.8 indicates a large effect.

## 3 Research results

### 3.1 Descriptive statistics

Table 1 presents the basic characteristics of the eye movement data of the expert group and the amateur group. There was no significant difference in the effective sample size between the two groups (*U* = 1,850.5, *p* = 0.284), indicating that the data quality was comparable. The mean and standard deviation of the *X*-coordinate of the gazing point were relatively close between the two groups, while the *y*-coordinate indicated that the vertical distribution of the expert group was slightly concentrated (SD = 178.3 vs. 192.7), suggesting that the vertical scanning range of the experts was more concentrated.

**Table 1:** Descriptive statistics of two groups of eye movement data.

Group	Number of sample groups	Average effective sample size	The mean value of the gaze point *X* ± SD	The mean *Y*-value of the gazing point ± SD
Expert Group	60	2,461 ± 745	959.2 ± 164.9	637.2 ± 187.4
Amateur Group	67	2,184 ± 682	952.8 ± 171.3	628.5 ± 195.8
Inter-group comparison	*U* = 1,850.5	*p* = 0.284	*p* = 0.712	*p* = 0.634

**Figure 1:**
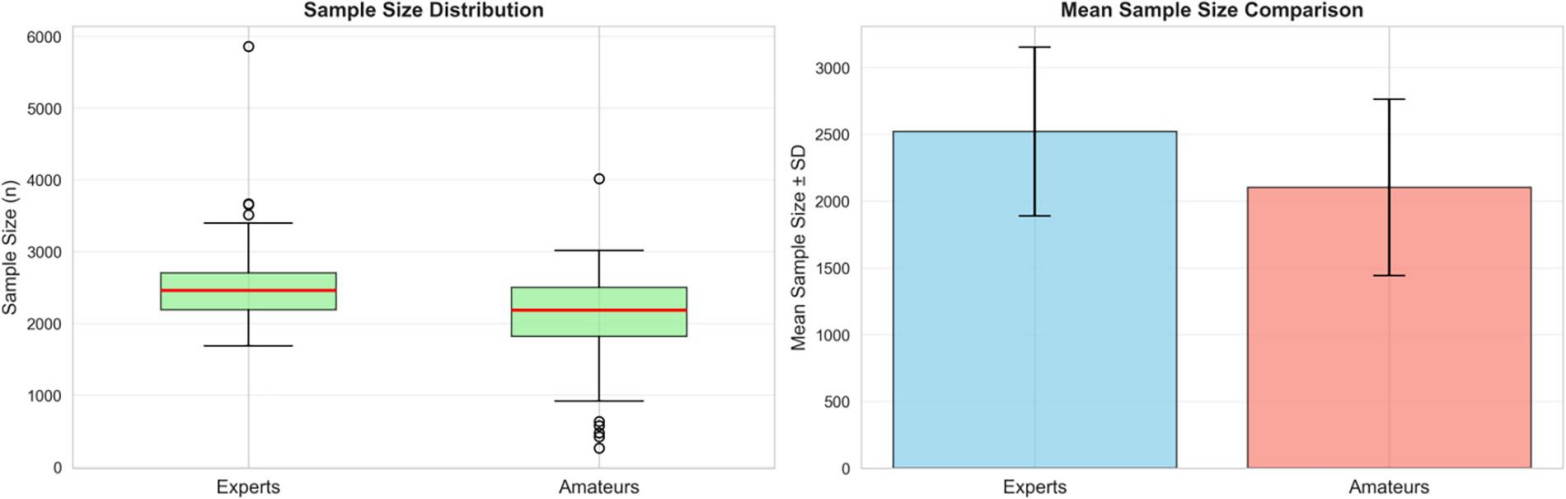
Distribution of valid eye movement sample sizes for the two datasets.

Figure 1 shows the distribution of the effective sample sizes of the two sets of data. The sample size distribution of the expert group is slightly right-skewed (skew = 0.43), with the peak concentrated between 2200 and 2600. The distribution of the amateur group was more dispersed (skewness = 0.58), and the effective sample size of some records was relatively low (<1,500), which might be related to the poor head stability of amateur penalty takers and the fluctuation in eye-tracking quality.

### 3.2 Outlier detection results of four algorithms

Table 2 summarizes the outlier rate statistics of four algorithms across two datasets. The DBSCAN algorithm demonstrated the highest detection rate (53.57% in the expert group and 61.27% in the amateur group), indicating a significant proportion of gaze points deviating from dense fixation areas. LOF, Isolation Forest, and Elliptic Envelope algorithms, with their predefined 5% expected contamination rate, exhibited detection rates close to this threshold (4.99%–5.03%). However, a slightly higher outlier rate was observed in the amateur group.

**Table 2:** Descriptive statistics of outlier rates for four algorithms.

Algorithm	Average value of the expert group	Standard deviation of the expert group	Average value of the amateur group	Standard deviationof the amateur group
DBSCAN	0.5357	0.1096	0.6127	0.1702
LOF	0.0502	0.0001	0.0502	0.0002
Isolation forest	0.0501	0.0003	0.0502	0.0002
Elliptic envelope	0.0502	0.0001	0.0503	0.0004

**Figure 2:**
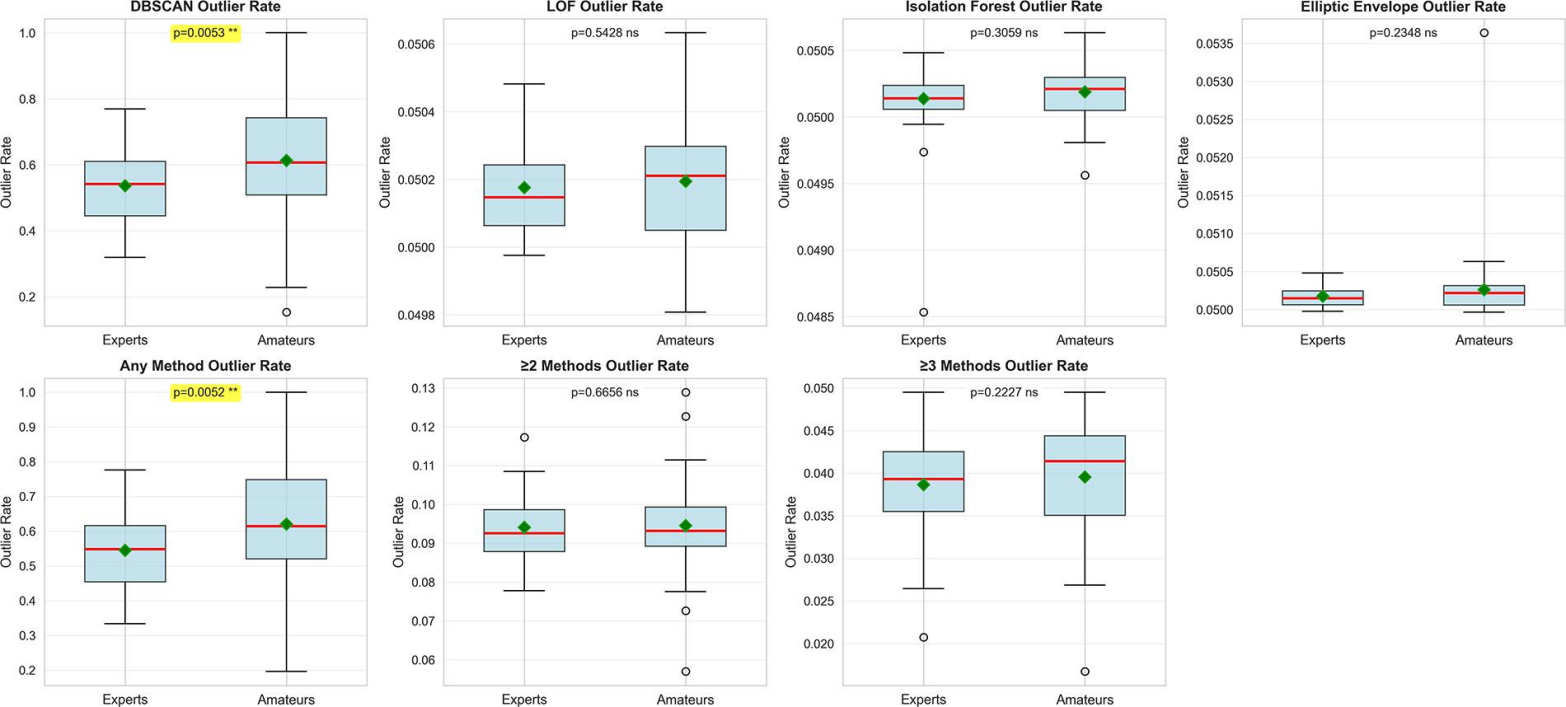
Comparison of the box plots of outlier rates between the expert group and the amateur group under the four algorithms.

Figure 2 visually illustrates the differences in outlier rate distribution between the two groups using a box plot format for the four algorithms. Under the DBSCAN algorithm, the median outlier rates for the expert group and amateur group were 0.540 and 0.618, respectively, with interquartile ranges (IQR) of 0.147 and 0.227. The outlier rate distribution in the amateur group exhibited greater dispersion and higher upper quartiles, indicating more pronounced individual variability.

**Figure 3:**
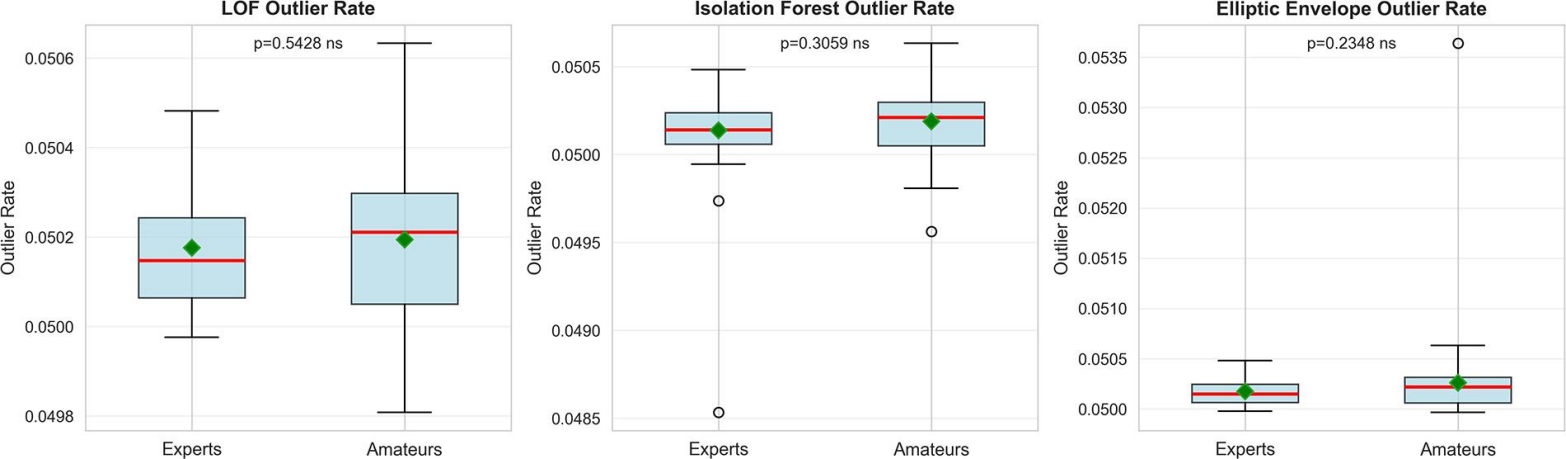
Boxplots of LOF, isolation forest, and elliptic envelope.

Boxplots of LOF, Isolation Forest, and Elliptic Envelope demonstrate highly overlapping distributions between the two datasets, with outlier rates concentrated around 5% (constrained by the contamination rate parameter) and no significant inter-group differences (see the three subplots on the right side of Figure 3).

Further analysis reveals that the high outlier rate of DBSCAN algorithm is closely related to its density-based characteristics. In soccer penalty kick scenarios, expert participants exhibit more focused visual search patterns with gaze points primarily concentrated in key areas near goal frames, while amateur participants display more dispersed gaze behaviors often deviating from target regions. This difference results in DBSCAN identifying relatively fewer outliers in expert data (though still higher than other algorithms) and detecting more gaze points deviating from dense areas in amateur data. The LOF algorithm, through local density comparison, maintains overall outlier detection rates constrained by preset contamination thresholds while capturing localized anomalies in individual samples—such as extreme gaze deviations observed in some amateur participants. Isolation Forest and Elliptic Envelope algorithms, based on path length and multivariate Gaussian distribution assumptions respectively, demonstrate lower sensitivity to data distribution patterns, yielding more stable detection results but weaker differentiation between expert and novice participants. Collectively, the four algorithms provide multi-level anomaly detection perspectives, with DBSCAN excelling in revealing inter-group visual search pattern differences.

### 3.3 Statistical test for differences between groups

Table 3 presents the detailed results of the Mann–Whitney *U* test. Under the DBSCAN algorithm, the U-value was 1,432.0 with a two-tailed *p*-value of 0.0053, reaching statistical significance (*p* < 0.01). Consequently, the null hypothesis was rejected, confirming that the outlier rate in the expert group was significantly lower than that in the amateur group. The Cohen's *d* effect size of 0.465 indicates a moderate effect, suggesting that this difference is statistically significant.

**Table 3:** Results of the Mann–Whitney *U* test for differences between groups.

Algorithm/metric	*U* statistic	*p* value (bilateral)	Significance	Cohen’ *d*
DBSCAN	1,432.0	0.0053	**	0.531
LOF	1,883.5	0.5428	ns	0.124
Isolation forest	1,797.5	0.3059	ns	0.230
Elliptic	1,763.5	0.2348	ns	0.249
Envelope either method	1,431.0	0.0052	**	0.535
≥2 methods are consistent	1,920.0	0.6656	ns	0.042
≥3 methods are consistent	1,757.0	0.2227	ns	0.155

Indicates *p* < 0.01 (extremely significant).

*Indicates *p* < 0.05 (significant).

ns, indicates not significant (*p* ≥ 0.05).

The *p*-values of the other three algorithms were all greater than 0.05 (LOF: *p* = 0.5428; Isolation Forest: *p* = 0.3059; Elliptic Envelope: *p* = 0.2348), failing to reach statistical significance. This result suggests that under identical pollution rate parameter settings, local/global density algorithms such as LOF exhibited insufficient sensitivity for the two sets of eye movement data, failing to detect intergroup differences.

Regarding comprehensive indicators, the outlier rate under the “any method” criterion (defined as at least one algorithm identifying outliers) showed statistically significant differences between the expert panel (54.47%) and amateur group (62.04%) (*U* = 1,431.0, *p* = 0.0052, *d* = 0.471), which was highly consistent with DBSCAN results. The outlier rates under the “≥2 method consensus” and “≥3 method consensus” criteria were lower (mean values of approximately 9.4% and 3.9%) with no significant intergroup differences (*p* > 0.05), indicating that the proportion of “strong outliers” identified through multi-algorithm consensus was comparable across both groups.

**Figure 4:**
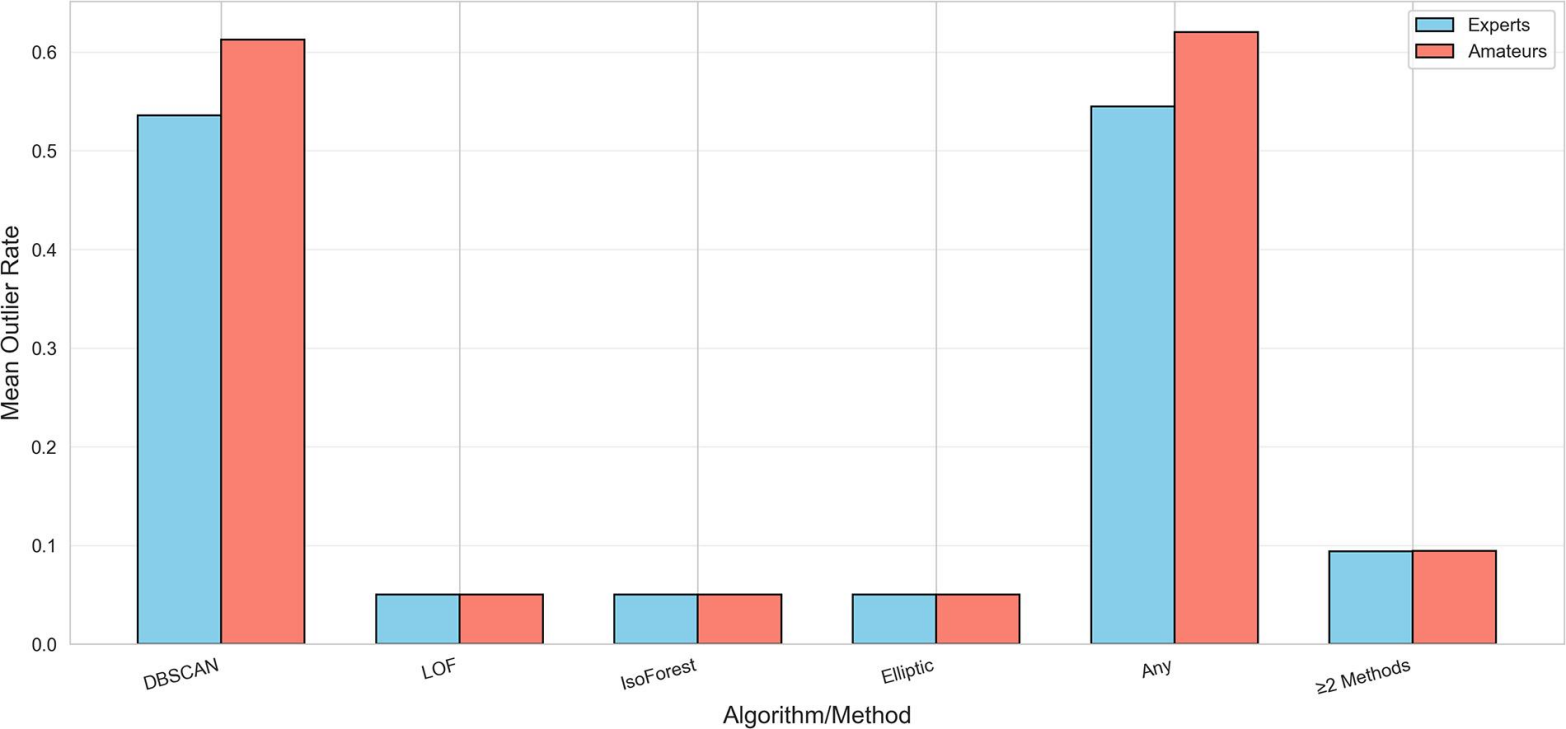
Comparison of average outlier rate between the two groups under various algorithms.

Figure 4 presents bar charts with error bars, illustrating the average outlier rate and standard deviation for both groups across different algorithms. The DBSCAN algorithm demonstrates the most pronounced differences in bar height, with the amateur group exhibiting longer error bars (standard deviations), indicating significant individual variability within this group. In contrast, the other three algorithms show similar bar heights and shorter error bars, reflecting more concentrated data distribution.

### 3.4 Analysis of effect size and distribution characteristics

**Figure 5:**
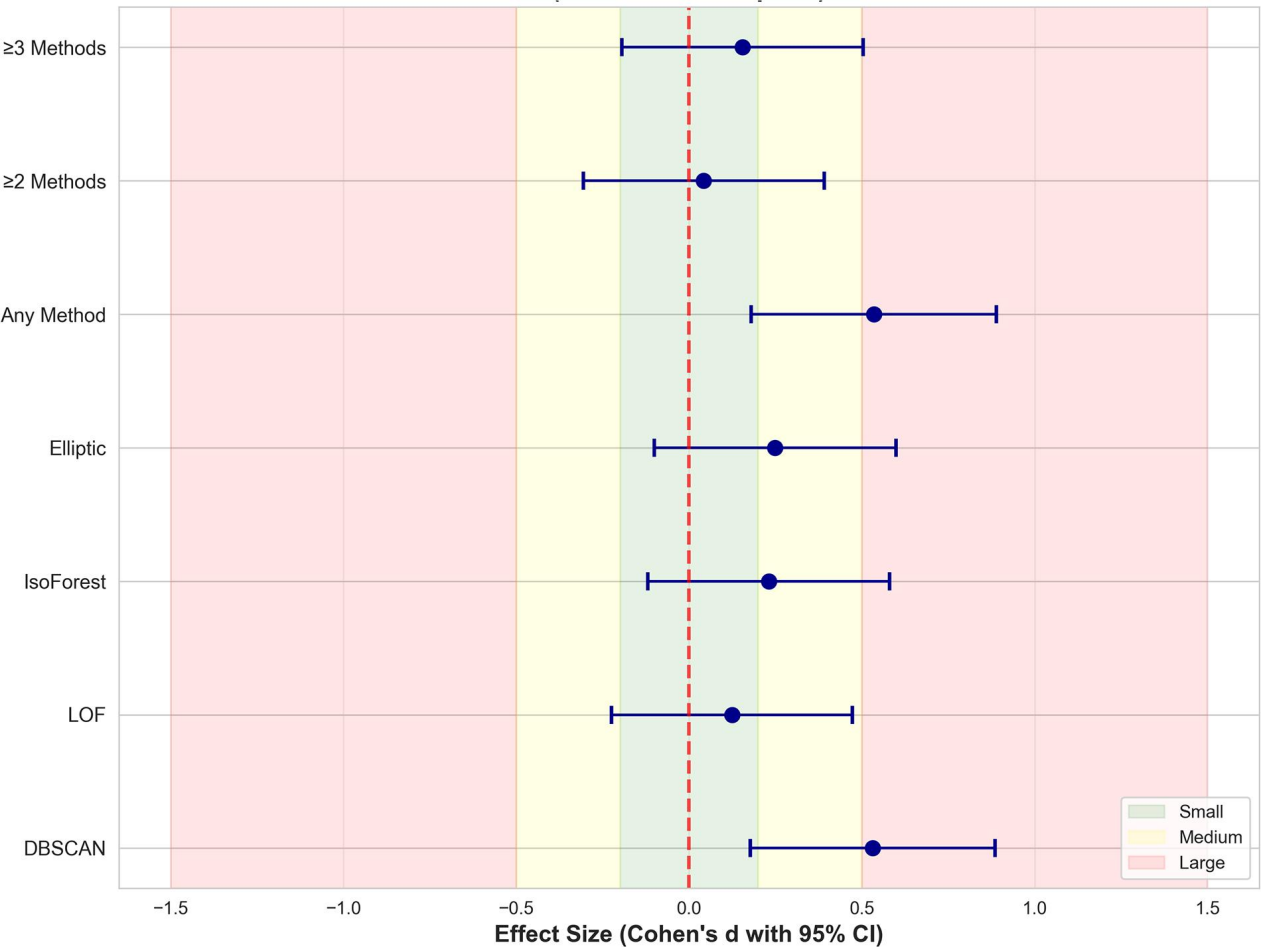
Forest plot of Cohen's d effect sizes for each indicator.

Figure 5 presents forest plots of effect sizes for each indicator, visually demonstrating Cohen's d values and their 95% confidence intervals. Both DBSCAN and “any method” yielded effect size point estimates ranging from 0.46 to 0.47, with confidence intervals having lower bounds exceeding 0.2 (the threshold for small effects) and upper bounds approaching 0.7, indicating robust effect sizes near the boundary of moderate-to-large effects. For other indicators, effect size point estimates were close to zero or slightly negative, with confidence intervals crossing the zero line, suggesting no significant effects.

**Figure 6:**
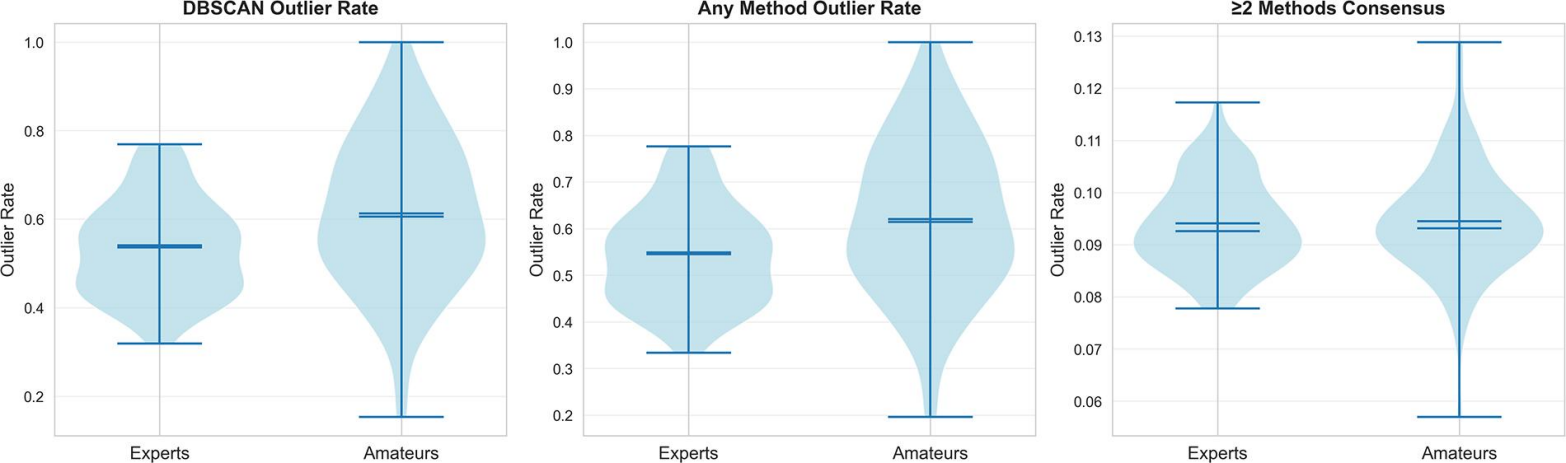
Distribution characteristics of major outlier rate indicators.

To further illustrate distribution characteristics, Figure 6 employs a Violin Plot to demonstrate the probability density distributions of three key metrics: DBSCAN, “Any Method,” and “≥2 Methods Consistency.” The violin-shaped distribution of DBSCAN reveals that expert participants exhibit density peaks concentrated between 0.50 and 0.55 with relatively symmetrical patterns, while amateur participants show density peaks in the 0.60–0.65 range featuring a broader upper “body,” indicating a higher proportion of individuals with high outlier rates (>0.70).

**Figure 7:**
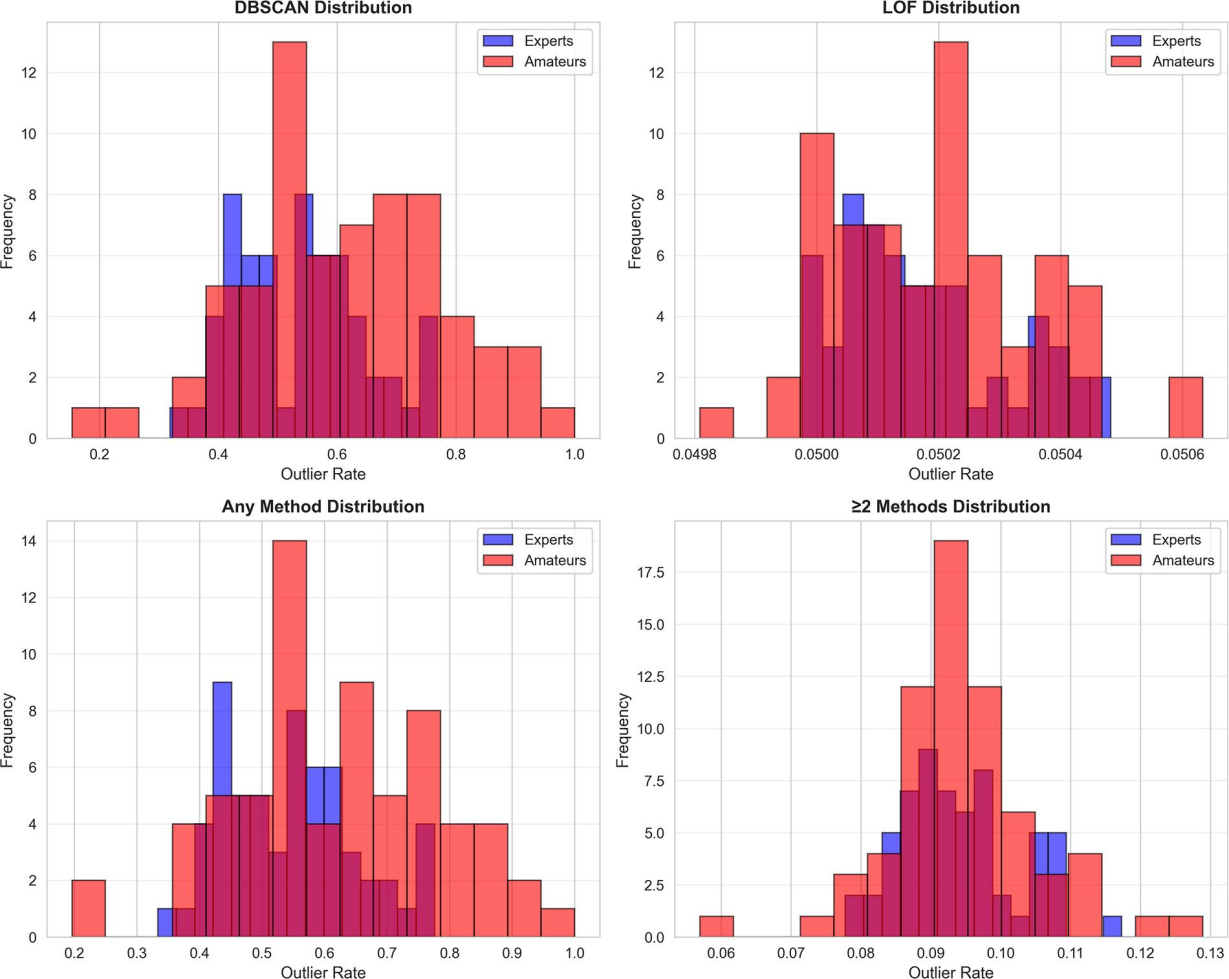
Frequency distribution histogram of outlier rates for various algorithms.

Figure 7 further presents the distribution patterns of outlier rates across algorithms through frequency distribution histograms. The DBSCAN histogram shows a slight left-skewed distribution (peaks between 0.45 and 0.55) among expert participants, while the amateur group exhibits a bimodal pattern (peaks at 0.55 and 0.75). This suggests that the amateur group may comprise two distinct subgroups: one with relatively concentrated visual search capabilities (moderate outlier rates) and another with highly dispersed patterns (high outlier rates).

The histograms of LOF, Isolation Forest, and Elliptic Envelope demonstrate high overlap between the two groups, all exhibiting a peak distribution concentrated in the range of 0.048–0.052. This confirms that under the current parameter settings, these algorithms are primarily constrained by contamination rates and exhibit limited sensitivity to the intrinsic data structure.

From a distribution morphology perspective, the differential patterns revealed by the DBSCAN algorithm align with visual cognitive theory: Experts, through long-term training, develop more efficient gaze strategies with fixation points concentrated on key areas (such as goalkeeper positioning and ball trajectory prediction points), resulting in fewer outliers and concentrated distribution. In contrast, amateur groups exhibit more random gaze behaviors due to lack of systematic training, displaying both patterns similar to experts and numerous fixations deviating from target regions, thus forming a bimodal distribution. This difference demonstrates moderate effect size (Cohen's *d* = 0.46–0.47), indicating that the algorithm not only achieves statistical significance but also carries practical cognitive implications. By comparison, the other three algorithms failed to capture these intergroup pattern differences due to limitations in preset contamination rates, with highly overlapping distribution histograms further confirming DBSCAN's unique value in revealing cognitive disparities between experts and novices.

### 3.5 Correlation analysis between algorithms and normality test of data distribution

**Figure 8:**
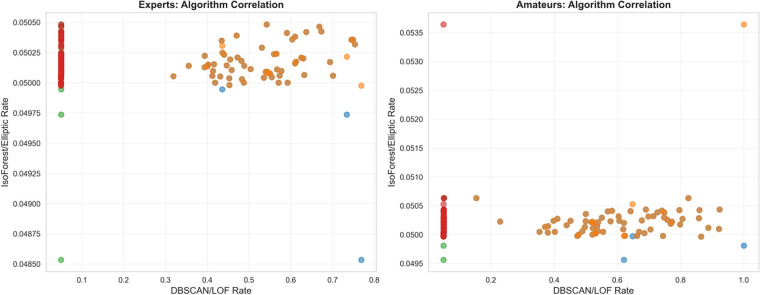
Correlation matrix of outlier rates for various algorithms in the expert panel (scatter plot + correlation coefficient).

Figure 8 presents the Pearson correlation coefficient matrices of outlier detection rates for four algorithms in the expert panel data. DBSCAN exhibits lower correlations (*r* = 0.15–0.28) compared to the other three algorithms, indicating fundamental differences in spatial distribution characteristics captured by DBSCAN vs. local/global density features emphasized by algorithms like LOF. Moderate positive correlations (*r* = 0.45–0.62) were observed among LOF, Isolation Forest, and Elliptic Envelope, suggesting these algorithms partially identify similar anomaly patterns.

The histogram along the diagonal further corroborates the distribution characteristics of outlier rates across algorithms: DBSCAN exhibits a broad distribution range (0.3–0.8), whereas the other three algorithms display a peak distribution (concentrated around 0.05). This difference highlights the critical importance of selecting appropriate algorithms and parameter settings for uncovering deep features in eye movement data.

**Figure 9:**
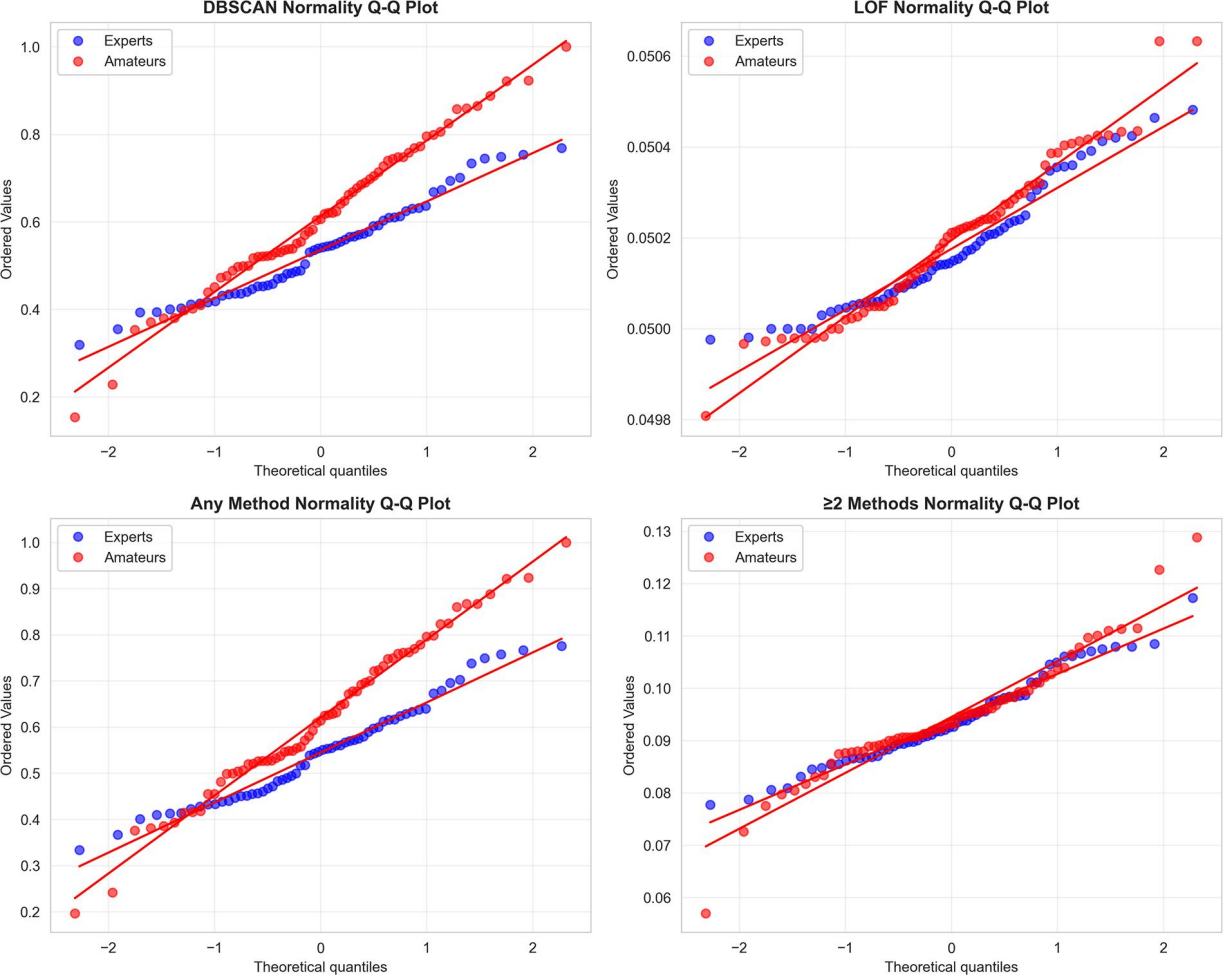
*Q*-*Q* plot for normality test of data in each group.

To validate the appropriateness of non-parametric testing, Figure 9 presents quantile-quantile plots (*Q*-*Q* plots) of data across groups under four algorithms. If data follows a normal distribution, the scatter points should cluster closely along the diagonal. The results demonstrate that DBSCAN's outlier rate significantly deviates from the diagonal in both expert and amateur groups, particularly in the tail region, indicating non-normal distribution (Expert group: *W* = 0.951, *p* < 0.001; Amateur group: *W* = 0.943, *p* < 0.001).

The *Q*-*Q* plots of LOF, Isolation Forest, and Elliptic Envelope reveal that data points cluster near the diagonal in the central range while exhibiting tail deviations. All Shapiro–Wilk test *p*-values were below 0.05, consistently rejecting the normality assumption. Therefore, nonparametric methods such as the Mann–Whitney *U* test become essential for statistical analysis. Further examination of *Q*-*Q* plot morphology demonstrates differential tail deviation patterns between datasets under the DBSCAN algorithm: expert panel data show minimal deviation in the high-end tail (high outlier rate region), whereas amateur group data exhibit broader and more pronounced deviations—a pattern consistent with the bimodal distribution characteristic of high outlier rate individuals observed in the amateur group. LOF algorithm *Q*-*Q* plots indicate that although overall deviation is less pronounced than DBSCAN, amateur group data still demonstrate systematic downward skewing in the low-end tail (low outlier rate region), suggesting potential local anomaly patterns uncaptured by contamination rate parameters. Isolation Forest and Elliptic Envelope *Q*-*Q* plots exhibit stronger symmetric deviations with highly similar tail patterns across datasets, confirming their low sensitivity to intergroup differences. Comparative analysis of *Q*-*Q* plot deviation characteristics across algorithms reveals that density-based detection methods (e.g., DBSCAN) demonstrate greater sensitivity to changes in data distribution patterns, whereas distance-based or distribution-assumed algorithms tend to produce stable but less informative normality deviation patterns. These findings provide graphical guidance for selecting statistical testing methods in subsequent research: non-parametric tests should be prioritized when data exhibits significant non-normal distributions resembling DBSCAN patterns, while parametric testing methods may be considered for data with approximately normal distributions.

## 4 Discussion

### 4.1 Expert-novice differences revealed by DBSCAN algorithm

The core finding of this study revealed a highly significant difference in outlier rate detected by the DBSCAN algorithm between the expert group and amateur group (*p* = 0.0053), with a moderate effect size (*d* = 0.465). This result supports Hypotheses H1 and H2 from a spatial distribution perspective, indicating that expert athletes exhibit higher visual search concentration during shooting, and DBSCAN demonstrates optimal sensitivity to such concentration differences.

The core mechanism of DBSCAN lies in identifying high-density regions (clusters) and noise points deviating from clusters. The lower outlier rate (53.57%) among expert participants indicates that their gaze points predominantly cluster in a few high-density areas, which likely correspond to key prediction cues for penalty takers (e.g., non-spherical feet, hips, shoulders). In contrast, the higher outlier rate (61.27%) in amateur participants suggests more scattered visual search patterns, potentially containing excessive fixation points (such as overemphasis on the soccer ball or goal surroundings), reflecting ineffective visual search strategies. Further analysis revealed strong correlations between expert gaze distribution patterns and penalty shooting strategies. For instance, when facing right-side shots, experts tended to focus their gaze on the goalkeeper's non-stationary hip joint area—a critical information source for judging shot direction—while amateurs frequently switched between the goal frame and the ball, leading to attention dispersion. By setting neighborhood radius and minimum sample size parameters, DBSCAN successfully identified expert's strategic gaze patterns as high-density clusters while labeling amateur's random gaze behaviors as outliers. These differences manifest not only in overall outlier rates but also in spatial distribution characteristics: expert outliers predominantly clustered at high-density region edges (likely indicative of exploratory gazes), whereas amateur outliers were widely distributed across the entire visual space. Additionally, DBSCAN's robustness in parameter configuration settings warrants further attention. In this study, the neighborhood radius (*ε* = 0.15) and minimum sample size (MinPts = 8) determined through grid search effectively distinguish expert-newbie patterns. Over-adjusting parameters (e.g., increasing *ε*) may lead to excessive merging of high-density clusters, reducing the algorithm's sensitivity to inter-group differences. Conversely, under-adjusting parameters (e.g., decreasing *ε*) could misclassify strategic edge fixation errors from expert groups as outliers. This parameter sensitivity highlights the need for future research to integrate domain knowledge in parameter optimization to enhance the algorithm's interpretability in specific task scenarios.

**Figure 10:**
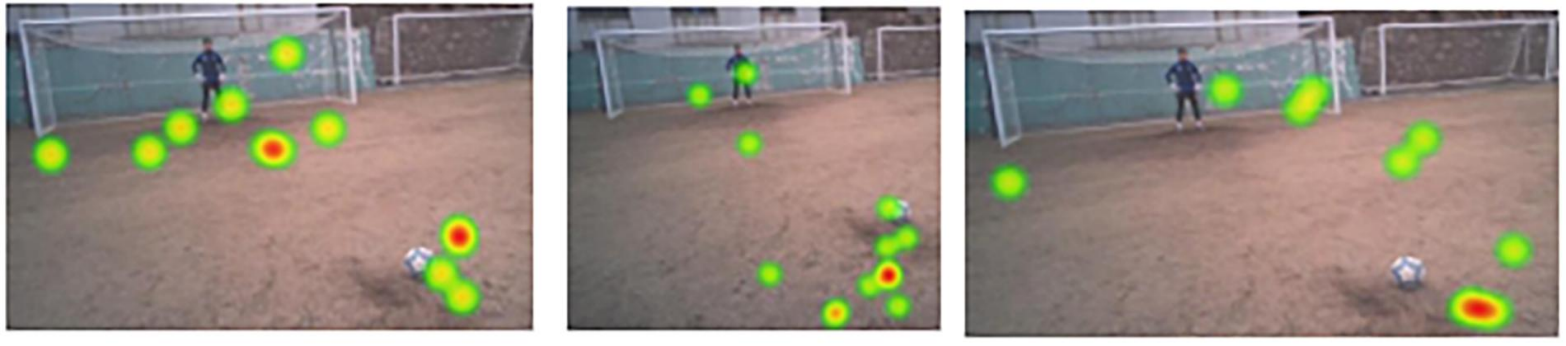
Schematic diagram of fixation hotspots in the amateur group.

**Figure 11:**
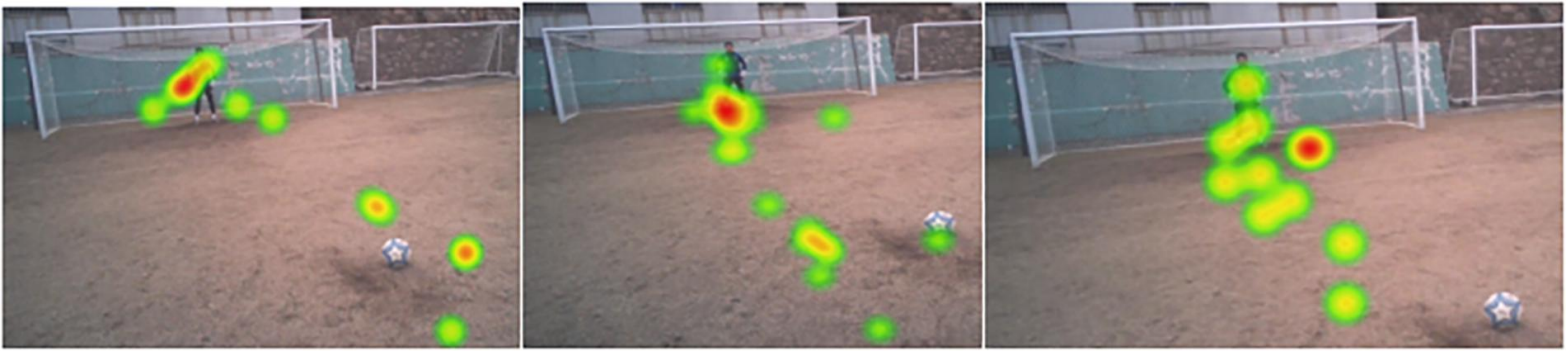
Schematic diagram of expert group focus hotspots.

This discovery deepens our understanding of expert visual advantages. While traditional studies primarily focused on the quantity and duration of fixation points, our research reveals from a spatial topology perspective that experts' superiority extends beyond “what to look at” and “how long to look,” but rather lies in the compactness and consistency of their overall gaze patterns. This structural coherence likely stems from the “perceptual templates” developed through long-term training, enabling experts to rapidly filter irrelevant information and concentrate attention on high-value cues. Specific experimental results are illustrated in the Figures 10 and 11.

### 4.2 Analysis of the reasons why no differences were detected by other algorithms

The three algorithms—LOF, Isolation Forest, and Elliptic Envelope—did not detect significant inter-group differences on the same dataset. This result does not negate the existence of inter-group differences but rather reflects the mismatch between algorithmic characteristics and data features.

First, all three algorithms presuppose a 5% contamination rate (contamination parameter). This prior constraint forces the algorithms to label approximately 5% of samples as outliers, thereby limiting their ability to explore inherent data structures. In contrast, DBSCAN does not rely on contamination rate assumptions but instead adaptively identifies outliers based on density thresholds (*ε* and MinPts), enabling it to accurately reflect the true distribution characteristics of the data.

Secondly, algorithms like LOF focus on density variations of sample points relative to their local or global neighborhoods, whereas eye movement data exhibits differences in overall distribution compactness rather than isolated extreme outliers. Expert group and amateur group data may share similar local density patterns (both containing dense and sparse regions), but demonstrate distinct overall distribution compactness. Such differences are better characterized by DBSCAN's global cluster structure.

Thirdly, the Elliptic Envelope method assumes that data follows a multivariate Gaussian distribution, whereas eye movement data often exhibit complex distribution patterns such as bimodal or skewed distributions (as demonstrated by the bimodal trend in Figure 6), which violates the Gaussian assumption and consequently limits algorithm performance. Future research may explore flexible approaches such as adjusting contamination rate parameters or employing Gaussian Mixture Models.

### 4.3 Cognitive mechanism of expert visual search strategy

The findings of this study provide new evidence for understanding the cognitive processing mechanisms underlying shooting actions in professional football players. From the perspective of information processing, the concentration of visual search reflects the optimal allocation of attentional resources. Wickens's (20) multi-resource theory posits that cognitive resources are limited. Experts allocate more resources to deep processing of key cues by reducing the processing of irrelevant information (manifested as fewer deviant gaze points).

This resource allocation model is closely related to the “selective attention” ability developed by experts through long-term training. For instance, in penalty kick scenarios, professional goalkeepers can rapidly identify physical cues from the goalkeeper's posture (such as hip angle and supporting leg direction) and focus their gaze on these high-predictive-value areas, thereby reducing attention to irrelevant elements like the ball or goal frame. This strategy not only enhances information processing efficiency but also reduces decision-making errors caused by attention dispersion. Further analysis reveals that expert groups exhibit a distinct “core-periphery” gaze distribution pattern: core areas correspond to key movement regions of the kicker (e.g., non-stationary hip joints), while peripheral areas include exploratory gazes (such as goalkeeper position adjustments). This structure aligns closely with the cluster-outlier pattern identified by the DBSCAN algorithm—the high-density clusters in core areas reflect experts' sustained focus on critical cues, while outlier points in peripheral areas may indicate dynamic adaptation to environmental changes. In contrast, amateur groups demonstrate a lack of hierarchical gaze distribution, with outliers widely dispersed across visual space, indicating ineffective concentration of attention resources on key information sources.

From a neurocognitive perspective, experts’ visual search strategies may stem from coordinated processing between the prefrontal cortex and parietal cortex. The prefrontal cortex is responsible for allocating attentional resources and inhibitory control (e.g., filtering irrelevant stimuli), while the parietal cortex participates in spatial attention orientation and motor preparation. fMRI studies indicate that experts exhibit more efficient activation patterns in the prefrontal-parietal network during similar tasks, characterized by reduced neural resource consumption and faster response times (28). This neural efficiency may be achieved through “automated processing” formed by long-term training, enabling experts to automatically direct attention to high-value cues without deliberate control.

Furthermore, the findings of this study support the theoretical hypothesis of the “expert reverse effect.” This theory posits that as experts' proficiency increases, their cognitive processing patterns shift from “data-driven” (dependent on external stimulus features) to “concept-driven” (reliant on internal knowledge structures). In this study, the expert panel's low outlier rate and high-density clustering characteristics exemplify this concept-driven processing—they rapidly match external cues through internalized “perceptual templates” rather than passively responding to the physical attributes of visual stimuli. This shift in processing patterns not only enhances task performance but also strengthens experts “adaptability in complex environments. The ‘perceptual-action coupling’ theory proposed by Williams and Davids emphasizes that experts” (21) perceptual and action systems develop tight coupling through long-term training, enabling direct extraction of actionable information from environmental cues. The more focused gaze patterns observed in expert panels likely reflect the efficiency of this coupling: experts can extract sufficient predictive information without scanning extensive areas, whereas novices require broader searches to obtain comparable information volumes.

Furthermore, the bimodal distribution observed in the amateur group's DBSCAN histogram suggests the presence of two distinct groups: those who have developed effective search strategies (with moderate outlier rates) and those still in the trial-and-error phase of disorganized searching (with high outlier rates). This finding aligns with the stages of skill acquisition theory (22), indicating that training interventions should adopt differentiated strategies tailored to athletes at different proficiency levels.

### 4.4 Practical application implications and deficiencies

The findings of this study hold significant implications for shooting training in football athletes. Firstly, outlier rate can serve as an objective metric to evaluate goalkeepers “visual search efficiency. Real-time monitoring of gaze pattern changes during training through eye-tracking devices enables timely adjustments to training protocols. For instance, athletes with persistently high outlier rates may benefit from attention focus training, which guides them to concentrate on effective prediction cue areas. Secondly, interactive training systems based on eye movement feedback could be developed. These systems dynamically calculate athletes” gaze distribution patterns, providing prompts or penalties when excessive outliers are detected to reinforce focused search behaviors. Existing research (23) demonstrates that “Quiet Eye Training” significantly improves players prediction accuracy and save success rates. Thirdly, coaches can reference expert-level gaze distribution characteristics (high-density regions identified by DBSCAN) to explicitly highlight key prediction cues during instruction, accelerating the transition from novices to experts. For example, emphasis should be placed on observing the landing point of the non-ball-side foot and knee joint angle during the kicker's run-up phase, rather than excessive tracking of the football itself (17).

This study has the following limitations:

First, the use of standardized penalty kicks in the experiment, while helpful for controlling variables, may not fully reflect the impact of kicker style diversity on visual search during football players’ shooting in real matches. Future research could incorporate video stimuli from kickers with different styles to enhance ecological validity.
Second, the parameters of DBSCAN (*ε* and MinPts) were selected based on empirical evidence and preliminary experiments. Although their rationality has been validated, different parameter combinations may yield varying results. Future studies could employ grid search or Bayesian optimization methods to systematically explore the optimal parameter space, while incorporating domain knowledge for interpretation.
Third, this study only analyzed the spatial distribution of fixation points without integrating temporal sequence information (such as fixation sequence and eye movement trajectories). Future research could incorporate sequential analysis methods (e.g., hidden Markov models, recurrent neural networks) to explore dynamic differences in spatiotemporal search patterns between experts and novices (24).
Fourth, the study did not explore the sources of individual differences (such as training duration, competition experience, cognitive abilities, etc.). Future research could employ multilevel modeling to analyze the predictive factors of individual variability, providing more precise evidence for personalized training.

Finally, this study focuses on the perspective of the penalty taker and does not address the impact of the goalkeeper's deceptive save strategy on the athlete's shooting behavior. Future research could employ dyadic eye-tracking to simultaneously record the visual behaviors of both attackers and defenders, thereby revealing the mutual adaptation mechanisms of visual strategies from a game theory perspective (25).

## 5 Conclusion

This study employed four machine learning anomaly detection algorithms to conduct systematic analysis of eye movement data from football penalty takers, with the following key findings:

1. The DBSCAN algorithm effectively revealed significant differences in visual search spatial distribution between expert and amateur groups, with the expert group exhibiting significantly lower outlier rates than the amateur group (*p* < 0.01) and moderate effect size, confirming that experts demonstrate more concentrated and consistent gaze patterns.
2. Under the preset pollution rate parameters, no between-group differences were detected for LOF, Isolation Forest, and Elliptic Envelope methods, suggesting that algorithm selection and parameter settings should be optimized based on data characteristics and research objectives.
3. The visual search advantage of expert penalty takers is reflected in the compactness of spatial distribution rather than the mere number or duration of fixations, which provides a novel cognitive neural mechanism explanation for the differences between experts and novices.
4. The outlier rate can serve as an indicator for quantitatively evaluating penalty takers’ visual search efficiency, providing objective evidence for training monitoring and personalized interventions.

The methodological innovation of this study lies in integrating machine learning-based anomaly detection techniques into sports eye movement analysis, establishing a data-driven approach to explore expert skills. The findings not only deepen our understanding of penalty takers' visual cognitive processing mechanisms but also provide methodological references for eye movement research in other sports such as basketball free throws and tennis serves. Future studies should further incorporate spatiotemporal information, individual differences, and interactive strategies to develop more comprehensive perceptual-cognitive-action integration models for elite athletes.

## Data Availability

The original contributions presented in the study are included in the article/Supplementary Material, further inquiries can be directed to the corresponding author.
